# Carotid shunt provides cerebral protection during emergency coronary artery bypass grafting in a patient with bilateral high grade carotid stenosis: a case report

**DOI:** 10.1186/1749-8090-6-33

**Published:** 2011-03-20

**Authors:** John K Bellos, Nektarios Kogerakis, Charalampos Kiriazis, Alexandros Gougoulakis, Matthew Panagiotou

**Affiliations:** 1 Department of Vascular Surgery, Athens Medical Center, 5-7 Distomou St, 15125, Marousi Athens, Greece; 2 Department of Cardiac Surgery, Athens Medical Center, 5-7 Distomou St, 15125, Marousi Athens, Greece

## Abstract

**Background:**

Management of patients with co-existent coronary and carotid disease is a controversial and challenging issue. The risk for stroke after coronary artery bypass grafting (CABG) in patients with hemodynamically significant carotid stenosis is up to 30%. In these patients a common practice is to proceed first with the restoration of cerebral perfusion and then perform the coronary revascularization. The rationale is that this strategy will reduce perioperative neurological morbidity and mortality. However, what happens when the carotid procedure is acutely complicated by cardiac instability which necessitates the interruption of the carotid procedure?

**Case report:**

We describe a case of a patient with unstable angina and high grade asymptomatic bilateral carotid stenosis who underwent emergency combined CABG and carotid endarterectomy (CEA). Due to hemodynamic instability, ST-T changes, hypotension and bradycardia, upon completion of endarterectomy we placed a carotid shunt and the patient was put on cardiopulmonary bypass through median sternotomy. After triple CABG (duration of 90 minutes) we concluded the interrupted CEA procedure with primary closure of the carotid arteriotomy with the shunt in place. The postoperative course was uneventful and the patient was discharged after a week. In extreme cases with bilateral severe carotid stenosis and coronary artery disease where the carotid procedure should be interrupted, we suggest the use of carotid shunt which can provide adequate cerebral perfusion giving time to cardiac surgeon to perform the life saving cardiac procedure first.

## Background

Management of patients with co-existent coronary and carotid disease is a controversial and challenging issue [1]. The risk for stroke after coronary artery bypass grafting (CABG), in patients with hemodynamically significant carotid stenosis is up to 30% [2]. Therefore, in these patients a common practice is to proceed first with the restoration of cerebral perfusion and then perform the coronary revascularization. The rationale is that this strategy will reduce perioperative neurological morbidity and mortality. However, according to our knowledge, there is no published data concerning combined carotid endarterectomy (CEA) and CABG where intraoperatively the carotid procedure was acutely complicated by cardiac instability necessitating the interruption of the carotid procedure. We describe our experience using a temporary carotid shunt in order to maintain cerebral perfusion until CABG was completed and then the operation was concluded with the closure of carotid arteriotomy.

## Case presentation

### Patient's history and management

**Figure 1:**
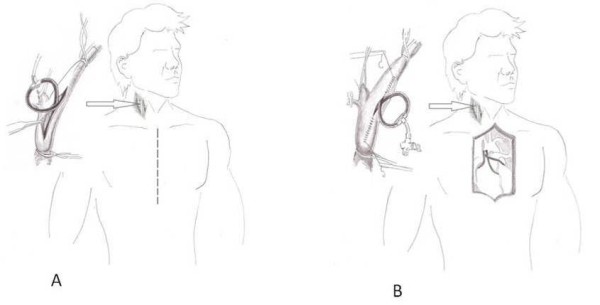
**Line drawings depict stages of the operative procedure:** A) Due to hemodynamic instability we stopped the carotid endarterectomy procedure, we placed a carotid shunt and we proceeded to emergency sternotomy, B) After the completion of 3 coronary artery bypass grafting and patient's weaning from cardiopulmonary bypass, we continued with the primary closure of carotid arteriotomy.

A 80 year old male patient with a history of coronary artery disease (CAD) and severe left ventricular dysfunction was urgently admitted in our institution with unstable angina. Ejection fraction was 20%. Coronary angiography revealed severe triple vessel disease. Moreover, duplex ultrasound which was performed urgently in intensive care unit (ICU), showed bilateral severe carotid stenosis (80-90% stenosis and unstable plaque in the right internal carotid artery and 70-80% stenosis in the left side). Both vertebral arteries were patent without reverse flow or any significant hemodynamic changes. The patient continued to be in unstable angina, therefore we decided to perform emergency combined surgery without a preoperative angiogram. After induction of general anesthesia the right carotid bifurcation was exposed by a standard lateral approach and simultaneously the left great saphenous vein was harvested. Immediately after the completion of endarterectomy and before starting the closure of arteriotomy the patient became hemodynamic unstable with ST-T changes, bradycardia and hypotension. Under these conditions we decided to interrupt the carotid procedure and place a Javid carotid shunt (Bard Peripheral Vascular Inc, AZ, USA), and immediately proceed with a median sternotomy and cardiopulmonary bypass (CPB) (Figure 1A). We performed an emergency triple CABG with saphenous vein grafts under extracorporeal circulation (ECC) and moderate hypothermia (28°C). After the patient's weaning from CPB and his pressure stabilization (90 minutes later) the CEA was completed with primary closure of carotid arteriotomy (Figure 1B). The patient was transferred to the ICU for one day and was discharged on the seventh postoperative day with improved left ventricular function and without neurological complications.

## Discussion

The optimal management of patients with combined carotid and coronary artery disease remains controversial. Various strategies have been proposed such as CEA alone, or CABG alone, or staged CEA and CABG, or staged carotid artery stenting (CAS) and CABG, and simultaneous CEA and CABG, or simultaneous CAS and CABG. In the majority of these approaches the CEA or CAS precedes the CABG because it seems that reduces the perioperative neurological morbidity and mortality. We report a case where the CEA procedure was interrupted by the CABG. The carotid procedure was finished after the completion of the CABG. All these handlings were possible through a temporary carotid shunt which proved sufficient to maintain adequate cerebral perfusion for 90 minutes. To our knowledge prolonged cerebral perfusion through a shunt during on pump emergency CABG in patients with bilateral severe carotid stenosis has not been previously reported.

Besides carotid artery stenosis, other stroke risk factors during CABG, frequently quoted in the literature, are: ascending aortic atherosclerosis, previous stroke or transient ischemic attack, age, hypertension, diabetes, smoking, peripheral vascular disease, left ventricular dysfunction, left main CAD, renal failure, and increased cardiopulmonary bypass time [3]. Our patient had the majority of these risk factors (age, left CAD, heavy smoker in the past, left ventricular dysfunction, peripheral arterial disease). The only preventive measure against stroke were the carotid shunt, which proved to be adequate, and the patent vertebral arteries.

In a recent case report the authors applied a 14 Fr cannula into the distal part of the internal carotid artery. A separate pump was connected in cannula and arterial blood at 23°C was delivered at a flow rate of 300 ml/min [4]. Although, based on classical findings, the normal carotid artery flow rate is 133-200 ml/min, the appropriate flow rate of the active cerebral perfusion is still unclear [5]. However, under CPB the cerebral autoregulation is severely impaired, thus a flow rate of 300 ml/min may result to hyperperfusion syndrome and cerebral hemorrhage. Moreover, differences between our technique and the previous mentioned are obvious. Our technique is less complicated, less expensive and less time consuming. Therefore, our report suggests that a carotid shunt could maintain cerebral perfusion and could provide cerebral protection for at least 90 minutes ECC in patient with high grade bilateral carotid stenosis.

Some surgeons prefer the off-pump CABG, especially in patients with severe carotid disease [6]. Off-pump CABG certainly has many advantages and in a recent study an aorto-carotid shunt was used in patients who underwent combined CEA and CABG with satisfactory results [7]. However, in our case this was not feasible because of patient's hemodynamic instability.

Finally, the important role of the shunt in the cerebral perfusion during CPB is enhanced by the fact that the patient was under diminished systemic pressure, moderate hypothermia and non pulsatile cerebral perfusion from the pump for 90 minutes.

## Conclusion

This case highlights the value of conventional carotid shunt to maintain intra-operative cerebral perfusion during emergency CABG in unstable patients with simultaneous carotid and coronary disease. We propose our technique as a bail-out trick in combined cases of CEA and CABG when the endarterectomy cannot be completed due to life threatening cardiac and hemodynamic instability.

## Consent

Written informed consent was obtained from the patient for publication of this case report. A copy of the written consent is available for review by the Editor-in-Chief of this journal.

## Competing interests

The authors declare that they have no competing interests.

## Authors' contributions

JB, AG, MP came up with original conception and design. JB, AG, MP, NK, CK reviewed the medical literature, and were major contributors in writing the manuscript. NK, CK formatted the media. All authors read and approved the final manuscript.
